# Mechanisms of drug resistance in nutrient-depleted colorectal cancer cells: insights into lysosomal and mitochondrial drug sequestration

**DOI:** 10.1242/bio.060448

**Published:** 2024-10-24

**Authors:** Serra Gülse Köse, Aliye Ezgi Güleç Taşkıran

**Affiliations:** Molecular Biology and Genetics Department, Baskent University, Ankara 06790, Turkey

**Keywords:** P-glycoprotein (P-gp/B1), Lysosomal drug sequestration, Mitochondrial drug sequestration, Colorectal cancer (CRC), Autophagy

## Abstract

This Review delves into the mechanisms behind drug resistance in colorectal cancer (CRC), particularly examining the role of nutrient depletion and its contribution to multidrug resistance (MDR). The study highlights metabolic adaptations of cancer cells as well as metabolic adaptations of cancer cells under low nutrient availability, including shifts in glycolysis and lipid metabolism. It emphasizes the significance of MDR1 and its encoded efflux transporter, P-glycoprotein (P-gp/B1), in mediating drug resistance and how pathways such as HIF1α, AKT, and mTOR influence the expression of P-gp/B1 under limited nutrient availability. Additionally, the Review explores the dual roles of autophagy in drug sensitivity and resistance under nutrient limited conditions. It further investigates the involvement of lysosomes and mitochondria, focusing on their roles in drug sequestration and the challenges posed by lysosomal entrapment facilitated by non-enzymatic processes and ABC transporters like P-gp/B1. Finally, the Review underscores the importance of understanding the interplay between drug sequestration, lysosomal functions, nutrient depletion, and MDR1 gene modulation. It suggests innovative strategies, including structural modifications and nanotechnology, as promising approaches to overcoming drug resistance in cancer therapy.

## Teaser

**Summary:** This Review examines drug resistance in colorectal cancer, focusing on the role of nutrient depletion in multidrug resistance. It highlights metabolic adaptations, and the role of MDR1/P-glycoprotein, and suggests innovative strategies to counteract resistance.

## Introduction

In 2020, more than 1.9 million incidences of colorectal cancer (CRC) were observed worldwide, ranking CRC as the third most common cancer. Additionally, 0.9 million deaths from CRC worldwide were recorded, ranking CRC as the second most common cause of cancer mortality (Morgan et al., 2023). For the past three decades, the first-line treatment of patients with CRC comprises of systemic chemotherapy involving 5-fluorouracil (5-FU), leucovorin, and oxaliplatin (Akhtar et al., 2014; Chionh et al., 2017). Despite new screening strategies and ongoing therapeutic developments, CRC is still one of the leading causes of cancer-related deaths (Sung et al., 2021). Nevertheless, the success of chemotherapy is restricted by drug resistance or low sensitivity (Silva and Gatenby, 2010). Therefore, it is particularly important to improve our understanding of basic processes such as the metabolism of cancer cells to gain insights into the divergent mechanisms they employ to provide survival and resistance mechanisms.

Several CRC cell lines are known for their ability to express characteristics of mature intestinal cells, such as enterocytes or mucus-producing cells (Simon-Assmann et al., 2007). This aspect adds a layer of complexity and relevance to their utilization. Hence, the CRC cell lines with the capacity to spontaneously differentiate, such as Caco-2 and T84 cell lines, are commonly employed as *in vitro* model systems to explore the mechanisms that control the differentiation-dedifferentiation processes of intestinal epithelial layer, to understand the causes of malignant growth, and find new therapeutic targets (Devriese et al., 2017). T84 cells in particular gain importance since they have the capacity to spontaneously differentiate into colonocytes, which are the epithelial cells of the colon and therefore provide important aspects of colon differentiation processes (Devriese et al., 2017).

CRC cell lines are integral to the field of cellular biology, serving not only as *in vitro* models that elucidate the processes of intestinal differentiation and malignant transformation, but also as unique systems that facilitate comprehensive investigations into the mechanisms underlying drug resistance (Cao et al., 2019). Beyond their primary application, CRC cell lines have contributed to understanding multidrug resistance (MDR), a phenomenon that poses challenges to the effectiveness of chemotherapeutic agents (Zhang et al., 2019).

The concept of MDR originates from the observation of cancer cells displaying resistance not only to a specific group of drugs, but also to a range of structurally and functionally unrelated drugs (Zhang et al., 2019). This fascinating phenomenon, observed across various cancer types, has prompted researchers to embark on an in-depth exploration of the intricate mechanisms contributing to this formidable obstacle in cancer treatment (Zhang et al., 2019).

The elucidation of drug sequestration within critical cellular organelles, including lysosomes and mitochondria, represents a landmark progression in our comprehension of the mechanisms underlying drug resistance. The study of Gotink et al. has revealed that CRC cell line HT-29 employes a strategic confinement tactic, entrapping drugs within such organelles (Gotink et al., 2011). This orchestrated drug entrapment acts to shield the cells from the detrimental effects of therapeutic agents (Gotink et al., 2011). An *in vitro* exploration into cellular dynamics elucidates that macroautophagy, a prominent cellular degradation process, plays a central role in this mechanism (Aguilera et al., 2012).

## Metabolic adaptations in nutrient-depleted CRC cells

Cancer cells are highly proliferating cells requiring the activation of both anabolic and catabolic pathways to generate macromolecules – such as proteins, lipids, and nucleic acids – necessary for cell proliferation and meet cellular energy requirements at the same time (Schmitt et al., 2007). Most of the cancer cells within a tumor have a limited availability of nutrients, which drives them to rewire their metabolism, therefore altering several aspects of cancer cell growth, survival, and response of cells to chemotherapy agents (Scumaci et al., 2020). These adaptations involve intricate mechanisms to acquire and utilize essential nutrients, illustrating the complex interplay between cellular metabolism and cancer progression (Eng et al., 2010). Although alterations in metabolic pathways in cancer cells were reported nearly a century ago, the rewiring of metabolic mechanisms has been only recently recognized as an emerging hallmark of cancer (Zielinski et al., 2017).

### Warburg effect and glycolytic shift

**Fig. 1.**
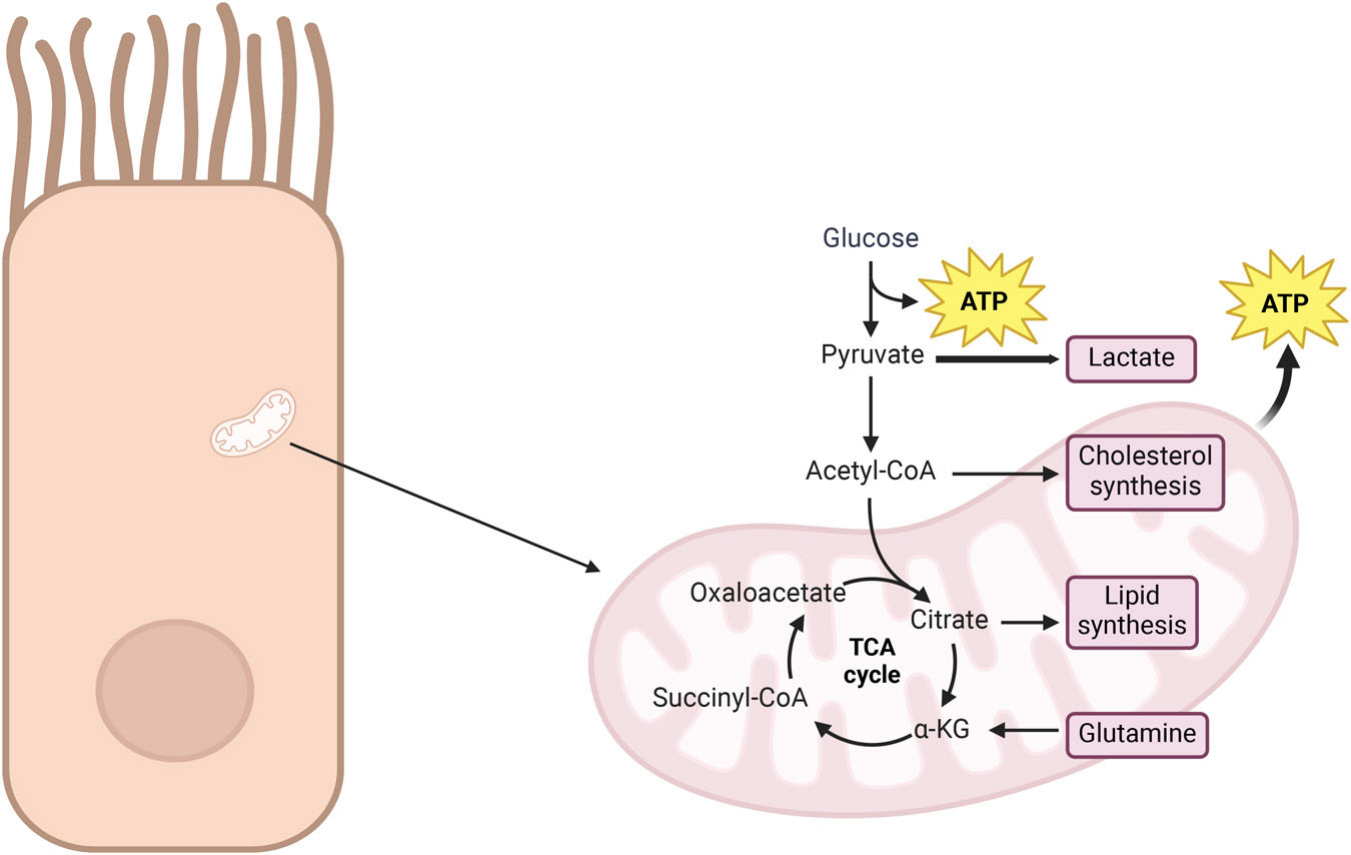
**The metabolic flexibility of cancer cells highlighting the Warburg effect, where glucose is preferentially converted into lactate despite the presence of oxygen, is emphasized in the diagram.** Such a metabolic shift allows for rapid tumor expansion by supplying energy and essential biosynthetic precursors. Key intermediates of the TCA cycle are detailed, including the conversion of pyruvate into acetyl-CoA and the role of citrate in lipid and cholesterol synthesis. Cancer cells exploit this flexibility, relying on either glycolysis or the TCA cycle for energy production and biosynthesis to sustain their rapid growth, as demonstrated in the figure. The figure was generated by using BioRender.com (“Stimulated metabolic activity”).

Warburg observed a unique trait in cancer cells compared to healthy ones. In the controlled *in vitro* environment of experiments, cancer cells demonstrate an increased utilization of glucose, transforming it into lactate, even in the presence of oxygen. This phenomenon is called the Warburg effect (Sun et al., 2019). According to Warburg, this metabolic shift is crucial for the transformation of normal cells into fast-growing cancer cells by affecting their usual method of respiration (Fig. 1). Unlike normal cells, cancer cells prefer the usage of glucose in the generation of building blocks to meet their proliferative demands (Icard et al., 2018). Arrest of glycolysis by over production of ATP and citrate is prevented via the downregulation of tricarboxylic acid (TCA) cycle. Citrate is mainly consumed to produce acetyl-CoA, which in turn is used in lipid biosynthesis and/or protein acetylation (Icard et al., 2018). Decreased mitochondrial activity also modulates reactive oxygen species levels to be in a concentration range compatible with cell proliferation (Icard et al., 2018). While most cancer cells use respiration for tumor growth, some tumors can grow using the TCA cycle without actively respiring. This flexibility highlights how cancer cells can adapt to different energy strategies (Sun et al., 2019).

### Glycolysis and lipid metabolism

To sustain rapid proliferation, cancer cells, especially in late-stage CRC, demonstrate a remarkable shift towards high glycolytic activity, engaging in anaerobic fermentation of converting glucose to fructose. This metabolic reprogramming is pivotal, given the estimated substantial energy requirement of approximately 17,700 kcal over 3 months to support metastatic CRC patients (Lieffers et al., 2009). The preference for glycolysis not only serves as a prominent energy source but also facilitates the generation of metabolic intermediates crucial for anabolic processes, including nucleotide synthesis and fatty acid production, as demonstrated by *in silico* analyses (Peng et al., 2018).

### Fatty acids and lipogenesis

**Fig. 2.**
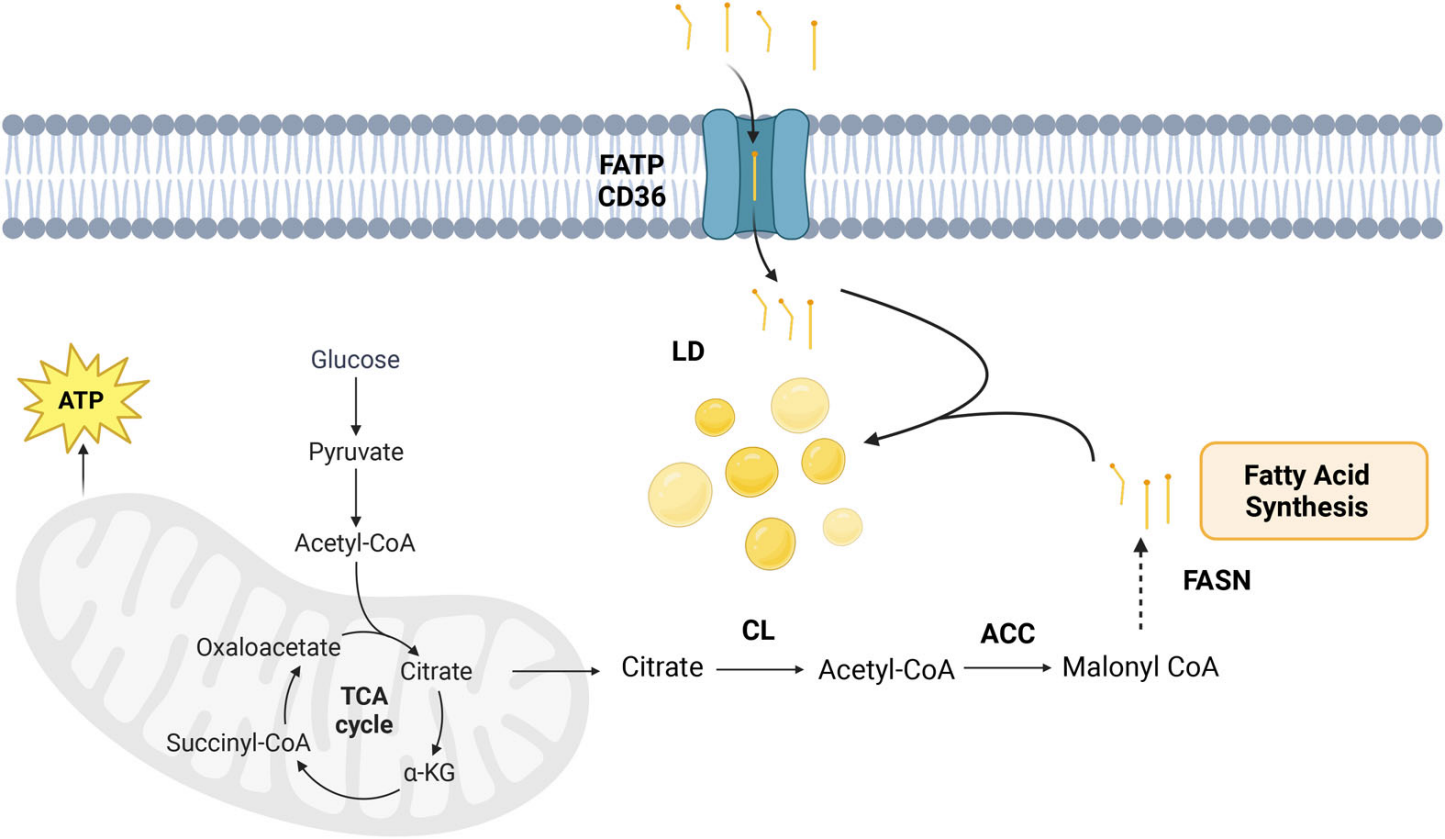
**The dysregulated process of *de novo* fatty acid synthesis in CRC cells.** Enhanced glucose metabolism drives the production of acetyl-CoA, which feeds into the TCA cycle. Citrate is then exported to the cytoplasm, where CL catalyzes its conversion back into acetyl-CoA, a precursor for fatty acid biosynthesis. Key enzymes such as ACC and FASN drive this process, providing essential lipid components for cell membrane formation and energy storage, thereby supporting cancer cell proliferation and progression. The figure was generated by using BioRender.com (“Metabolic pathway in mitochondria”). LD, lipid droplets; CL, citrate lyase; ACC, acetyl-CoA carboxylase; FASN, fatty acid synthase; FATP/CD36, fatty acid transporters.

Dysregulation of fatty acid metabolism in CRC cells serves as a significant energy source and, therefore, can contribute to various anabolic processes essential for cancer progression (Ni et al., 2017). Transforming cells (*in vitro* and *in vivo*) exhibit elevated *de novo* fatty acid biosynthesis, a hallmark characterized by increased activity of lipogenic enzymes, such as ATP citrate lyase (CL) and fatty acid synthase (FASN) (Bueno et al., 2019). Elevated free fatty acids can be stored in lipid droplets to be used as an energy source or in membrane synthesis (Cruz et al., 2020). Additionally, increases in fatty acid uptake through elevated expression of fatty acid transporters such as FATP/CD36 provides free fatty acid supplies for the cells *in vitro* (Schneider et al., 2014). The process of TCA cycle leading to *de novo* fatty acid synthesis is explained in Fig. 2. Elevated lipogenesis through such a mechanism is fundamental for synthesizing new membranes, supporting the formation of lipid rafts for enhanced cell growth, receptor signaling, and generating signaling molecules crucial for activating proliferative pathways, notably the protein kinase B (AKT) pathway, as observed *in vitro* transforming cell lines and *in vivo* mouse models (Ni et al., 2017). Concurrently, fatty acids are catabolized through β-oxidation, revealing the intricate balance in lipid metabolism within cancer cell lines (Ni et al., 2017). Additionally, a high level of fatty acid availability has been shown to promote metastatic processes by providing an energy supply to support invasion and colonization, prevention of immune surveillance from detecting tumor cells, and promotion of cancer cell survival via enhanced antioxidative mechanisms (Corbet et al., 2020; Qiao et al., 2020; Wu et al., 2019). SW480 and SW620 cell lines were well excepted as matched primary and metastatic CRC cell lines, since they were isolated from the primary colorectal tumor and its lymph node metastasis from the same patient, respectively. Our previous work indicated that L-glutamine starvation leads to enhanced free fatty acid levels in SW620 cells compared to SW480 cells. In contrast, the levels of free fatty acids were comparable when cells were grown with L-glutamine. The primary source of the enhanced fatty acid levels was observed to be fatty acid uptake rather than enhanced synthesis. Moreover, enhanced fatty acid levels contributed towards a higher motility accompanied by loss of cell-cell contact only in SW620 cells. These results point out the possible role of fatty acids in the metastatic behavior of CRC cell lines, as well as the metabolic plasticity of CRC cell lines compensating for the retrieval of specific groups of nutrients (Güleç Taşkıran et al., 2024b).

### Nutrient depletion protocols in CRC cell lines

In the tumor microenvironment, nutrient limitation is a common phenomenon due to the irregular and often insufficient blood supply to the tumor core (Pan et al., 2022). This results in a heterogeneous distribution of nutrients and oxygen, creating regions within the tumor where cells experience significant nutrient deprivation (Pan et al., 2022). A variety of nutrient starvation or limitation protocols are applied *in vitro* to mimic the tumor microenvironment. These protocols help researchers study how cancer cells, including CRC cell lines, adapt to the nutrient scarce conditions typical of the tumor core.

Treatment of CRC cell lines with HBSS or EBBS, which depletes all sources of nutrients, is one of the most applied protocols (Alves et al., 2015; Lauzier et al., 2019). It has been shown that treatment of CRC cell lines with EBSS/HBSS can alter the autophagy flux, and different CRC cell lines exhibit differential metabolic adaptations due to their high heterogeneity (Alves et al., 2015; Lauzier et al., 2019). Complete depletion of serum, glucose, and all or some of the specific amino acids is one of the commonly applied types of nutrient limitation protocol used as well (Ji et al., 2021; Li et al., 2021; Zhao et al., 2021). It has been shown that serum starvation results in synergistic effects on CRC cell lines when combined with chemotherapy agents, and this synergistic behavior was attributed to changes in the transcription of genes responsible for cell metabolism and cancer's stress pathways (Cherkasova et al., 2023). Another study has shown that long-term serine deficiency affects cell proliferation by regulating transcriptional coactivator YAP, showing the importance of serine/glycine metabolism in cell proliferation regulation (Zhao et al., 2021).

The cells at the core of solid tumors have lowered access to nutrients and oxygen compared to cells at the periphery (Pan et al., 2022). While many cells undergo cell death, some are likely to survive and possess metabolic adaptations to nutrient-deficient conditions (Pan et al., 2022). Considering the low (but not non-) availability of nutrients, another approach is to apply a restricted amount of nutrients to the cells rather than complete depletion. Several CRC cell lines have been shown to enhance the expression of one or more epithelial and mesenchymal markers, suggesting the activation of hybrid/partial EMT upon incubation in a medium containing 10% of glucose, glutamine, and serum found in a complete medium (Hüsnügil et al., 2024). This study shows how CRC cell lines can activate adaptive mechanisms that help them survive and even become more motile to escape challenging metabolic conditions (Hüsnügil et al., 2024).

### Cell-cycle regulators and oncogenic signaling

In the context of cancer, cell-cycle regulators play a pivotal role in maintaining a high rate of cellular growth (Lynch et al., 2012). Oncogenic signaling pathways, including AKT and mammalian target of rapamycin (mTOR), play a direct role in shaping glucose metabolism, boosting nutrient uptake, and promoting macromolecular biosynthesis to sustain uncontrolled cellular proliferation (Johnson et al., 2010). These pathways coordinate a metabolic shift marked by heightened glycolysis and suppressed oxidative metabolism, aligning with the energy requirements of cancer cells.

### Autophagy and mitophagy

Devenport et al. discovered that even under nutrient-rich conditions, CRC cell lines demonstrate a substantial reliance on autophagy for growth *in vitro*, as evidenced by a marked reduction in growth upon inhibition of autophagy (Devenport et al., 2021). This underscores the adaptability of cancer cells to employ alternative mechanisms for nutrient acquisition, a critical aspect for proliferation even in the presence of abundant nutrients.

Mitophagy plays a vital role in nutrient replenishment and cell maintenance in tumors, as demonstrated by research conducted both *in vitro* and *in vivo* animal models (Devenport et al., 2021). Several studies have explored how mitophagy contributes to drug resistance. Yin et al. conducted a study with 90 CRC patients and reported altered PINK1 immunoexpression in both primary and liver metastasis lesions resected (Yin et al., 2021). Additionally, lesions of liver metastasis predict a worse prognosis with abnormal expression, possibly due to the abnormal function of mitophagy (Yin et al., 2021). Ke et al. conducted further investigations on more mitophagy related proteins and reported abnormal expression of PINK1, TOMM22, and TOMM40 in CRC using 51 normal samples versus 616 CRC patient samples (Ke et al., 2024). Tang et al. highlighted that multiple proteins engage various pathways to activate mitophagy, promoting proliferation and survival in both CRC patients and *in vivo* animal models (Tang et al., 2023). Since mitophagy has gathered attention in recent years, it is still a complex mechanism that needs further studies to fully explain its involvement to cancer progression.

### Role of HIF, AKT, and mTOR in CRC metabolic plasticity

The interaction between hypoxia-inducible factor 1-alpha (HIF1α), AKT, and mTOR pathways is essential for regulating glycolysis, involving both the transcriptional upregulation and phosphorylation of key enzymes and transporters. Oxygen availability is a critical factor for cellular survival, and the tumor microenvironment's hypoxia-induced changes profoundly influence glycolytic enzymes and nutrient transporters (Ho et al., 2015). Dysregulation of hypoxia response mechanisms resonates across various cancers, impacting crucial aspects such as oxygen transport, angiogenesis, and immune cell function (Su et al., 2024). Under hypoxic conditions, cells undergo a metabolic shift from O_2_-dependent mitochondrial ATP production to O_2_-independent glycolysis (Ho et al., 2015). This transition is accompanied by heightened oxidative stress, nutrient deprivation, and endoplasmic reticulum stress (Zhang et al., 2020). Certain metabolites derived from the TCA cycle, including succinate, fumarate, and α-ketoglutarate, function as oncometabolites (William et al., 2021). These substances contribute to tumor growth by promoting oncogenic signaling, particularly through the upregulation and stabilization of HIF-1α, despite glycolysis being the favored metabolic pathway (Ho et al., 2015).

**Fig. 3.**
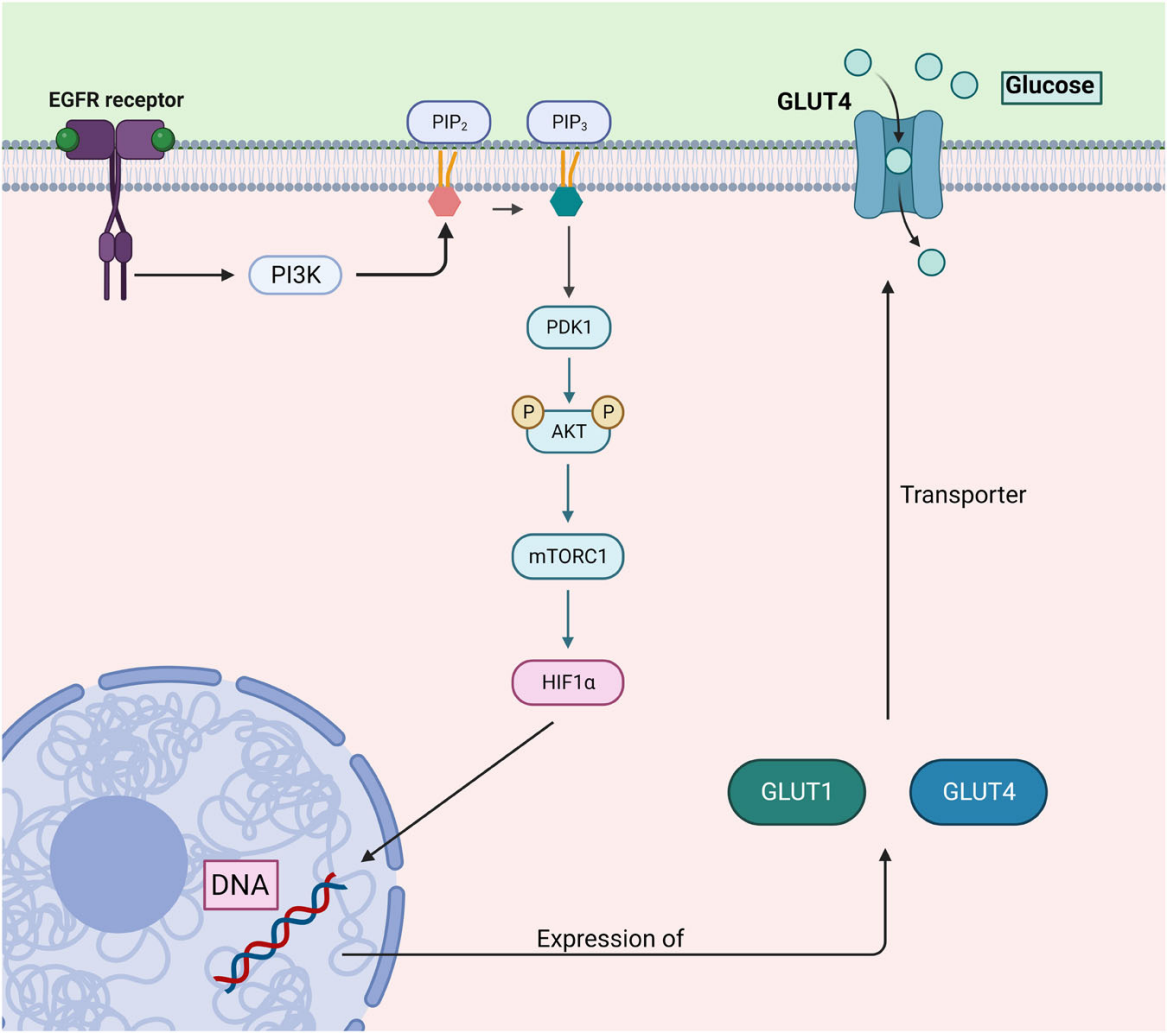
**This illustration details the PI3K/AKT/mTORC1/HIF1α pathway, focusing on its regulatory role in glucose uptake through GLUT1 and GLUT4 transporters.** Activation of the EGFR receptor triggers PI3K signaling, leading to the phosphorylation of AKT via PDK1. AKT activation promotes downstream signaling such as mTORC1 and the stabilization of HIF1α, which regulates the expression of GLUT1 and GLUT4 transporters. These transporters facilitate glucose uptake to meet the energy demands of cancer cells, further enhancing glycolytic metabolism. Dysregulation of this pathway is associated with CRC, where aberrant mTORC1 signaling and HIF1α stabilization contribute to tumor growth and metastasis through enhanced glucose uptake and metabolic reprogramming. The figure was generated by using BioRender.com.

Furthermore, mTOR signaling is often disrupted in tumors, significantly impacting cellular growth and metastasis through complex pathways, such as AKT-dependent cytoskeletal remodeling (Hervieu et al., 2020; Qian et al., 2004). Aberrant PI3K/AKT/mTORC1 signaling can be seen in a variety of excised tumors of CRC patients (Lai et al., 2014). AKT promotes glucose uptake through glucose transporter protein type 1 (GLUT-1) and glucose transporter protein type 4 (GLUT-4) (Wieman et al., 2007). It exerts influence on metabolic processes by impacting downstream transcription factors responsible for metabolic control, including HIF1α (Guru et al., 2015) (Fig. 3). Additionally, AKT triggers lipogenesis by directly phosphorylating and activating ATP-citrate lyase, resulting in elevated cytoplasmic acetyl-CoA levels (Bauer et al., 2005). Furthermore, AKT enhances the uptake of L-glutamine through SLC5A1, a process mediated by Myc. AKT also stimulates the flow of glucose into the oxidative pentose phosphate pathway through the downstream effector mTORC1 (Düvel et al., 2010). mTORC1 further activates this pathway by triggering the activation of SREBP and the upregulation of glucose-6-phosphate dehydrogenase (Yecies, 2012).

In brief, the above-mentioned pathways equip CRC cells with metabolic plasticity, which can confer survival advantages under nutrient-scarce conditions and, therefore, are highly actively modulated based on cellular needs.

## MDR1 encodes P-glycoprotein, a key contributor to developing MDR

MDR in cancer cells, often mediated by the overexpression of P-gp/B1, encoded by the MDR1 gene, poses a significant challenge in chemotherapy (Halder et al., 2022). P-gp/B1, a member of the ATP-binding cassette (ABC) superfamily, is expressed on various cellular membranes, including the plasma membrane and Golgi membrane (Gericke et al., 2022). It is widely present in normal human tissues, including the liver, kidney, colon, adrenal gland, intestine, placenta, hematopoietic precursor cells, and endothelial cells at various barriers (Gericke et al., 2022).

P-gp/B1 is a key efflux pump that protects tissues from harmful agents and foreign substances such as xenobiotics (Zhou, 2008). The upregulation of P-gp/B1 in drug-resistant tumors significantly impacts the efficacy of anticancer drugs, leading to treatment failures, as it pumps out a range of anticancer drugs (Liu-Kreyche et al., 2019). This heightened expression in cancer cells confers MDR, making these cells resilient against the therapeutic effects of various drugs (Liu-Kreyche et al., 2019). The molecular mechanisms regulating MDR are multifaceted, involving the human ABC transporters, breast cancer resistance protein (BCRP/ABCG2), and multidrug resistance-associated proteins (MRP1-2/ABCC1-2) (Zaja et al., 2008). These efflux transporters interact with various anticancer agents, minimizing intracellular exposure to cytotoxic drugs and contributing to MDR (Liu-Kreyche et al., 2019).

Not only many efflux proteins are involved in MDR, but also various regulatory mechanisms are involved in the expression of P-gp/B1. *In vitro* research findings on K562 leukemia cells reveal that the upregulation of P-gp/B1 expression involves a two-step process: mRNA stabilization and relief from translational block (Yagüe et al., 2003). Unlike transcriptional activation, this upregulation in K562 cells is attributed to an increase in mRNA stability, a specific phenomenon for MDR1 mRNA (Yagüe et al., 2003). The complexities of how long mRNA lasts, especially its short lifespan in unmodified K562 cells, highlight the complicated system that controls how the MDR1 gene is turned on or off (Yagüe et al., 2003). Incubation of T84 CRC cells in a nutrient limited condition *in vitro* shown to be resulted in the survival of a population of cells with high viability, accompanied by reduced sensitivity to chemotherapeutic agents such as 5-FU or doxorubicin (Güleç Taşkıran et al., 2024a). The mechanism by which the cells lose chemosensitivity is attributed to lysosomal trapping of chemotherapy agents, but an increased mRNA level expression of MDR1 gene or mRNA stabilization was also observed in the nutrient depleted cells, suggesting the presence of multiple mechanisms for drug resistance (Güleç Taşkıran et al., 2024a). Considering the existence of starvation stress responsive element in MDR1 gene promoter, elevated MDR1 gene expression is a plausible observation (Tanimura et al., 1992). Understanding these molecular nuances provides valuable insights for potential therapeutic interventions targeting P-gp/B1 mediated MDR in CRCs.

## Autophagy's impact on drug sensitivity: enhancing and inhibiting dynamics

Autophagy, a cellular process crucial for maintaining cellular homeostasis, exhibits dual roles in the context of cancer progression (Lock et al., 2011). In the early stages of tumorigenesis, autophagy acts as a tumor suppressor by degrading potentially harmful agents, damaged organelles, and misfolded proteins. It also maintains genomic stability by slowing damage repair, reduces chronic inflammation in the microenvironment, and limits the accumulation of reactive oxygen species (Degenhardt et al., 2006; Mathew et al., 2007). Under nutrient depletion conditions, autophagy plays a pivotal role, and induction of autophagy is regulated by factors such as AMP-activated protein kinase (AMPK) activation and mTORC1 inhibition (Yuan et al., 2013). In response to nutrient restriction (via the activation of AMPK and/or inhibition of mTORC1), autophagy is induced and serves pivotal roles for cell survival (Priault et al., 2010; Yang et al., 2014).

Several studies exhibited the role of autophagy in the proliferation of CRC cell lines under no and/or low nutrient availability. Zhang et al. exhibited that serum starvation-induced autophagy leads to the increased stability of LINC01615 (long non-coding RNA), which in turn promote the survival of CRC cell lines under serum-deprived conditions via pentose phosphate pathway (PPP) activation (Zhang et al., 2023). Tan et al. reported that Jumonji domain-containing protein 2B (JMJD2B) is involved in the promotion of CRC cell lines' survival upon glucose deprivation via enhancing the intracellular amino acid levels (Tan et al., 2020).

Autophagy serves as the primary mechanism for housekeeping, involving the recycling of redundant proteins and damaged organelles in normal cells. This process provides a survival advantage to tumor cells during tumorigenesis as exemplified by several studies mentioned (Gil et al., 2020; Lauzier et al., 2019). Nevertheless, if autophagy persists in a prolonged and excessive manner, it can unexpectedly lead to autophagic cell death. This contributes to the elimination of damaged or stressed cells, thereby enhancing the effectiveness of anticancer drugs (Su et al., 2024).

mTOR emerges as one of the main modulators of autophagy, influencing its dynamics and outcomes. Autophagy, acting as a guardian in drug resistance in cancer cell lines, supports cancer cell metabolism by recycling damaged components, preventing DNA damage, and inducing cancer drug resistance (Jung et al., 2009). This complex relationship is evident in various mechanisms, such as autophagy-regulated DNA damage response, autophagy-induced drug efflux (Liu et al., 2019; Orlotti et al., 2012).

**Fig. 4.**
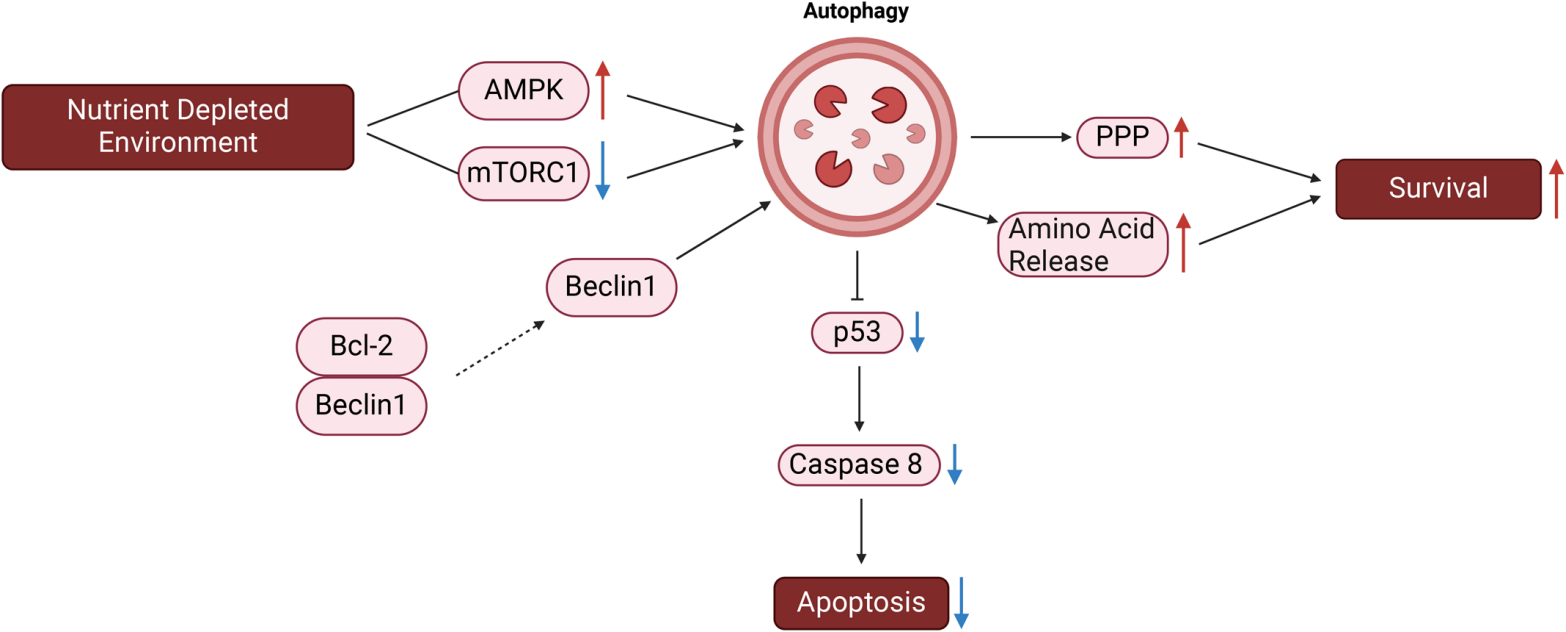
**This figure depicts how autophagy is activated in response to nutrient deprivation, playing a pivotal role in promoting cell survival and inhibiting apoptosis.** Under nutrient stress, autophagy is initiated through AMPK activation and mTORC1 inhibition. Once autophagy is induced, it supports cell survival by enhancing amino acid release and stimulating the pentose phosphate pathway, which sustains metabolic processes. At the same time, autophagy suppresses apoptosis by reducing p53 activity and degrading active caspase 8, a key component of the mitochondrial apoptosis pathway. Beclin1, a core autophagy protein, is essential in initiating and regulating the autophagic process by interacting with various proteins to promote autophagosome formation. The disruption of the Beclin1-Bcl-2 complex further enhances autophagy, contributing to cell survival, particularly in CRC cells. This regulation allows cancer cells to evade apoptosis, leading to tumor progression and increased resistance to therapies. The figure was generated by using BioRender.com. PPP, pentose phosphate pathway.

Furthermore, autophagy's involvement in drug resistance exceeds the nutrient stress, it is involved in the changes in apoptotic and survival signals regardless of the nutrient availability as well. Autophagy can degrade active caspase 8, a mitochondrial apoptosis pathway component, and exhibit a complex relationship with the tumor suppressor the tumor protein P53 (p53) (Hou et al., 2010). The disruption of the Beclin1/Bcl-2 complex and alterations in proapoptotic factors, antiapoptotic effectors, and survival signals contribute to autophagy-mediated drug resistance (Yang et al., 2020). Fig. 4 summarizes the involvement of autophagy induction in cancer cell survival and death.

Apart from its role as a guardian, autophagy can act as an executioner in tumor drug resistance (Gao et al., 2010). Autophagic cell death, induced by sustained autophagy, represents an alternative cell death approach when apoptosis fails, especially in drug-resistant tumors (Zhu et al., 2018). The intensity of oncogenic Ras signaling, and multiple activated signal pathways play crucial roles in determining whether autophagy acts as pro-survival or pro-death mediator (Elgendy et al., 2011).

Understanding the intricate interplay between autophagy, drug sensitivity, and nutrient availability provides valuable insights into developing targeted therapeutic strategies for overcoming drug resistance in various cancers.

## Lysosomes and drug sequestration leads to drug resistance

Lysosomes are highly acidic cellular organelles and the lysosomal reservoir of the cells serves pivotal roles along with autophagy in cell survival when nutrients are scarce (Paquette et al., 2021). Lysosomes, integral components of the endolysosomal system (ES), have conventionally been perceived as the cell's hub for recycling and waste management, which in turn are involved regeneration of building blocks (Nguyen et al., 2017). Recently, lysosomes have been also recognized as a signaling hub of the cells as they integrate both external and internal nutritional information to sustain cellular homeostasis (Mony et al., 2016). The orchestration of ES biogenesis and signaling through lysosomes primarily hinges on the activities of the transcription factor EB (TFEB) and mTORC1 signaling – a process frequently disrupted in the throes of oncogenic transformations (Li et al., 2016). The connection between mTORC1 and lysosomes is significant, with mTORC1 regulating V-ATPase expression among other functions (Deleyto-Seldas and Efeyan, 2021).

Lysosomes are present in all eukaryotic cells, and their morphology, size, and abundance are precisely regulated by the coordinated lysosomal expression and regulation (CLEAR) gene network (Palmieri et al., 2011). Key regulators, including TFEB, transcription factor E3 (TFE3), and mitochondrial translational initiation factor (MTIF), play crucial roles in activating this network (Palmieri et al., 2011). The regulation of these key regulators relies highly on mTORC1 activity. Under low nutrient availability, mTORC1 is inactive and released from the lysosomal surface which frees TFEB and TFE3 (Raiborg, 2018). These two TF promote the expression of CLEAR genes, which are involved in the lysosomal activity and autophagosomal degradation (Raben and Puertollano, 2016).

Lysosomes play diverse roles, including biomolecule discard, endocytosis, autophagy, exocytosis, and plasma membrane repair (Huynh et al., 2004; Machado et al., 2015; Marszalowicz et al., 2014). They are crucial for cell signaling pathways such as mTOR and AMPK (Wang et al., 2019). In cancer cells, lysosomes detoxify acidic metabolites generated by the Warburg effect, regulating abnormal pH gradients that impact proliferation, tumorigenesis, and drug resistance (Lozupone et al., 2015). Involvement of lysosomes in cancer survival is not limited to elimination of detrimental effects of the Warburg effect, but they also provide energy and building blocks under low nutrient availability conditions. Bandyopadhyay et al. identified a starvation response that allows cells to store the essential amino acid leucine within lysosomes to sustain protein synthesis *in vitro* (Bandyopadhyay et al., 2022). Degradation of extracellular proteins by lysosomes also provides cell cancer cells with survival advantages when nutrients are scarce (Pechincha et al., 2022).

In addition to providing building block and energy, lysosomes exhibit a unique ability to sequester lipophilic, weakly basic chemotherapeutic drugs through a non-enzymatic, non-transporter mechanism (Kazmi et al., 2013). Among these drugs, including doxorubicin, daunorubicin, vincristine, lapatinib, and nintedanib, many become trapped in lysosomes upon crossing the lysosomal membrane (Hurwitz et al., 1997; Kallus et al., 2018). Lysosomes act as reservoirs, pulling drugs from their target sites, but this trapping is reversible (Angus et al., 2008). The ionized drug can cross the lysosomal membrane by passive diffusion when cytosolic concentrations decrease (Kazmi et al., 2013). Lysosomal inhibitors such as chloroquine and bafilomycin A1 can also lead to release of entrapped drugs from lysosomes into the cytoplasm as well (Guo et al., 2016).

**Fig. 5.**
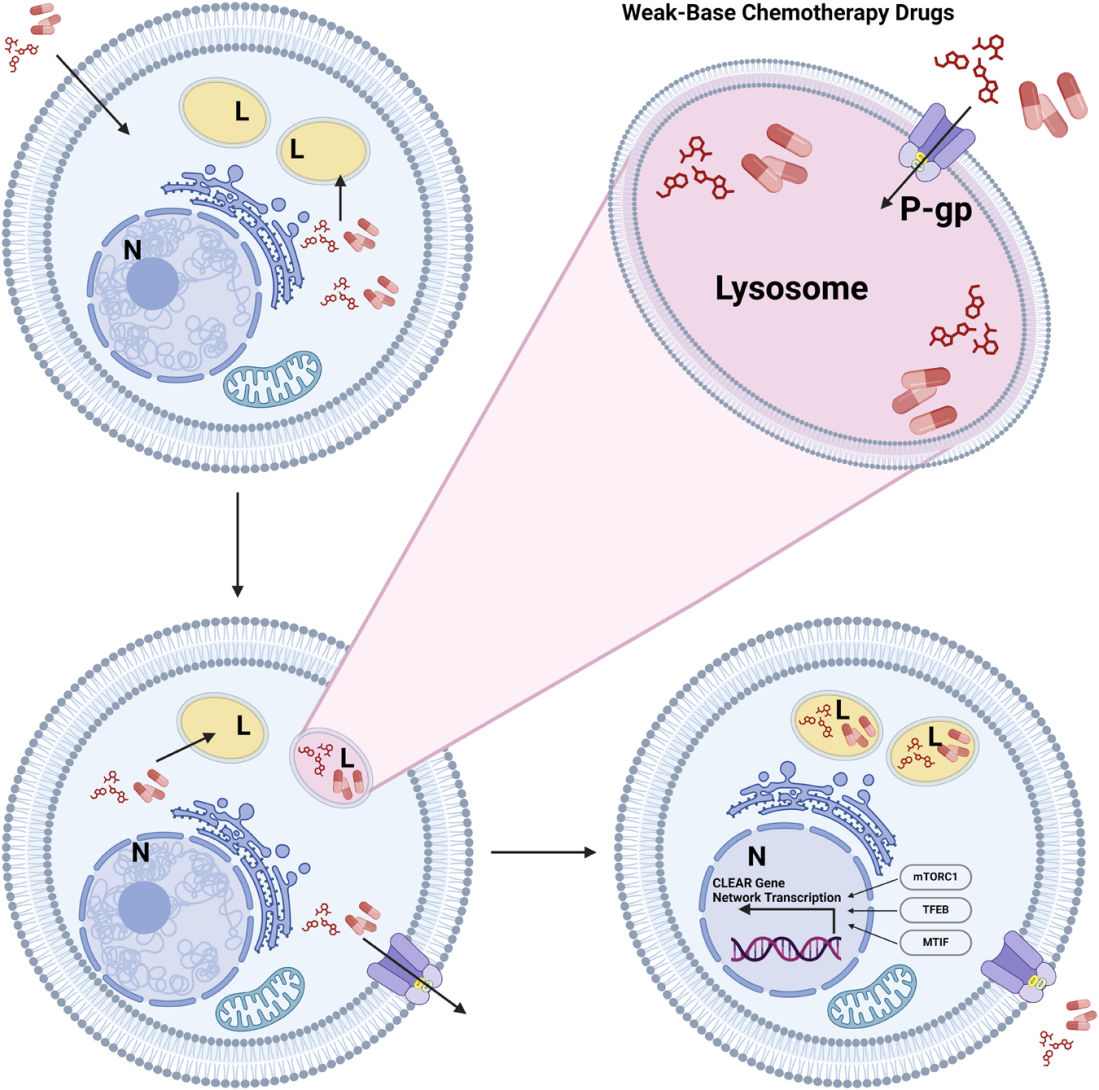
**This figure illustrates the process by which weak-base chemotherapy drugs become trapped in lysosomes, limiting their availability to cytosolic and nuclear compartments.** Once inside the cell, drugs enter lysosomes through passive diffusion and are further concentrated by the P-gp/B1 transporter located on the lysosomal membrane. This sequestration reduces the drugs' therapeutic efficacy at their target sites, such as the nucleus. Lysosomal biogenesis and function are regulated by the CLEAR gene network, controlled by transcription factors TFEB, TFE3, and MITF is also depicted in the figure. The CLEAR network governs lysosomal size, abundance, and enzymatic activity, particularly under the influence of mTORC1 signaling. In cancer cells, this system can become dysregulated, enhancing the lysosome's capacity to sequester drugs, which can contribute to drug resistance. The figure was generated by using BioRender.com. N, nucleus; L, lysosome.

TFEB, regulated by mTORC1 phosphorylation, influences the distribution of chemotherapeutic drugs between cytosolic and nuclear compartments (Cotter et al., 2015). Lysosomal entrapment reduces the therapeutic efficacy of these drugs at intended targets, such as nuclear DNA (Yaghini et al., 2017). Lysosomes also contribute to drug sequestration through ATP-binding cassette transporters (Merlos Rodrigo et al., 2019). P-gp/B1, a representative ABC transporter, is present on cell and lysosomal membranes (Colombo et al., 2014). Drugs, despite being distributed between cytosolic and nuclear compartments, unintentionally become entrapped in lysosomes, diminishing their therapeutic effects at intended target sites (Yaghini et al., 2017). Nutrient-limited T84 CRC cells showed increased MDR1 mRNA expression and reduced sensitivity to doxorubicin and 5-FU *in vitro*. Furthermore, these cells also exhibit decreased nuclear localization and increased lysosomal localization of doxorubicin, indicating that MDR1 may contribute to the drug's sequestration in lysosomes (Fig. 5) (Güleç Taşkıran et al., 2024a).

## Mitochondria and drug sequestration leads to drug resistance

As the powerhouse of the cells, mitochondria support metabolic flexibility and affect cancer progression by regulating proliferation, autophagy, and apoptosis (Liu et al., 2023). Mitochondria is a double membraned organelle with its own DNA (mtDNA) (Meeusen and Nunnari, 2003). Emerging from the mitochondrial matrix, these organelles have four main parts: the mitochondrial matrix, inner mitochondrial membrane (IMM), intermembrane space, and outer mitochondrial membrane (OMM) (Sesso et al., 2012). They are crucial for processes like energy generation, calcium signaling, lipid metabolism, ROS production, and apoptosis, and they are linked to conditions such as neurodegenerative disorders, diabetes, drug toxicity, and aging (Chen et al., 2003; Denecker et al., 2001; Juárez-Flores et al., 2020; Robb-Gaspers, 1998; Sala-Vila et al., 2016). As well as the mentioned pathologies, mitochondria and mitochondrial plasticity play important roles in cancer progression as well. Even though cancers cells have a profound reliance on glycolytic flux and lactic acid release (Warburg effect), mitochondrial plasticity is commonly observed in metastatic cancer cells (Parida et al., 2023). Thanks to mitochondrial plasticity, disseminating cells can shift from glycolysis to TCA cycle and fatty acid utilization in mitochondria to provide survival advantages in the host tissue (Parida et al., 2023).

Importance of mitochondrial plasticity has been exemplified in many studies. Since cancer cells are reliant on glucose metabolism for their high energy demand, the use of 2-deoxy-dglucose (2DG) is commonly applied to restrict glucose availability (Wu et al., 2024). Even though the use of 2DG is a logical way of cutting glucose supply, the metabolic plasticity of cancer cells enables alternative energy source usage. Pyruvate kinase M2 (PKM2) has been shown to localize to mitochondria under glucose starvation, with this localization mitochondrial membrane permeability was shown to be increased keeping mitochondrial functions active and enhance survival of HCT-116 CRC cells (Qi et al., 2019). Another study exhibits the importance of mitochondrial compensation under glucose starvation via usage of 2DG loaded nanoparticles and Ce6 (for mitochondrial activity inhibition) *in vitro* (Wu et al., 2024). LnNP@mSiO2-GC loaded with 2DG and Ce6 has been shown to both inhibit glycolysis (2DG) and impair mitochondrial activity (Ce6), in this way bypasses the compensatory pathway of the TCA cycle and enhanced the efficacy of 2DG single agent treatment (Wu et al., 2024).

In addition to the role of mitochondrial function under nutrient stress, mitochondrial vesicular formation of multivesicular bodies associated proteins (MAPS) appears to be a conserved process across all cell lines, irrespective of drug sensitivity (Abunimer et al., 2018). Notably, the efficiency of this process correlates with P-gp/B1 expression, conferring resistance and heightened survival rates in P-gp/B1 overexpressing cell lines (Abunimer et al., 2018). MAPS function as both drug sinks and extracellular transport vectors, indicating that enhanced MAPS production and drug sequestration correlate with heightened resistance in cancerous cell lines (Abunimer et al., 2018). Yet, directing interventions toward MAPS to counteract chemo-resistance in humans may present hurdles, potentially leading to lethal toxicity in normal cells (Abunimer et al., 2018).

Mitochondria, central to cellular metabolism and energy production, serves as the specific target of many chemotherapeutic agents, particularly through the mitochondrial apoptosis pathway (Guo et al., 2019). The presence and role of P-gp/B1 in the mitochondrial membrane have been subject to controversy. Recent studies have indicated that P-gp/B1 is expressed in mitochondria and functions in pumping drugs into the mitochondria (Guo et al., 2019). Additionally, the exchange of mitochondria between cancer cells and endothelial cells has been observed, contributing to chemoresistance in cancer cells (Guo et al., 2019). Pasquier et al. presented that ovarian and breast cancer cell lines were able to form tunneling nanotubes with stromal cells under same culture conditions, and transfer of mitochondria from stromal cells to cancer cell lines has been observed with mitochondrial specific dye MitoTracker (Pasquier et al., 2013). Moreover, sorting of mitochondria receiving and non-receiving cancer cells showed that acquisition of mitochondria enables cancer cells with chemoresistance (Pasquier et al., 2013). This observation implies that cancer cells have the capacity to communicate resistant traits via mitochondrial transport. Consequently, delving into the functional status and proteomics of mitochondria in cells exhibiting drug resistance is pivotal for unraveling the intricate mechanisms underpinning multidrug resistance.

## Nutrient sensing pathways and P-gp/B1 expression: insights into the PI3K/Akt/mTOR network's role in drug resistance

Navigating the intricate landscape of MDR reveals the paramount role of signaling pathways controlling P-gp/B1 expression (Yuan et al., 2020). Within this intricate web, the PI3K/Akt cascade stands out as a key regulator, acknowledged for its profound influence on P-gp/B1 expression (Yuan et al., 2020). Activated by phosphorylation, Akt initiates a sequence of events by phosphorylating inhibitor of nuclear factor kappa B (IκBα), which then causes IκBα to dissociate from NFκB. Subsequently, NFκB translocate to the nucleus, amplifying the expression of the MDR1 gene, the genetic blueprint for P-gp/B1 (Yuan et al., 2020).

Adding another layer of complexity, the downstream effector of Akt, mTORC1, plays a crucial role in nutrient sensing and MDR1 gene expression, as demonstrated in both *in vivo* and *in vitro* studies. Ma et al. demonstrated that inhibition of mTORC1 activity via Adriamycin treatment, reverses multidrug resistance in CRC cells, which is associated with increased autophagy, apoptosis and reduced MDR1 gene expression (Ma et al., 2015). Another study has been demonstrated the role of proton pump inhibitors (PPIs) in the expression of P-gp/B1 *in vitro* and *in vivo* (Chen et al., 2018)*.* Treatment of the multidrug-resistant SGC7901 gastric adenocarcinoma cell line with PPIs resulted in diminished levels of P-gp/B1 through the PI3K/AKT/mTOR/HIF-1α signaling pathway (Chen et al., 2018). These studies accentuate the nuanced interplay between PI3K/Akt, mTOR, HIF-1α, and P-gp/B1 in orchestrating drug resistance.

This intricate regulatory network extends its reach into glucose metabolism, where aberrant PI3K/Akt activation emerges as a hallmark of cancer aggressiveness and drug resistance. HIF-1α, a downstream target of PI3K/Akt, takes command over genes encoding crucial glucose metabolism mediators, including GLUTs (Zhang et al., 2014b). Further elucidating this network, conducted both *in vivo* and *in vitro* experiments with Nuciferine, a bioactive compound that inhibits the AKT/PI3K/ERK pathway (Qi et al., 2016). This inhibition suppresses the activation of nuclear factor erythroid 2-related factor 2 (Nrf2) and HIF-1α, subsequently reducing the expression of P-gp/B1 and BCRP in HCT-8/T and A549/T cell lines (Liu et al., 2020). The association between PI3K/Akt, mTOR, HIF-1α, GLUT, and P-gp/B1 reveals a sophisticated regulatory network that influences drug resistance mechanisms, offering potential targets for therapeutic intervention.

Understanding these pathways is essential for developing targeted strategies to overcome multidrug resistance in cancer cells.

## Strategies to overcome resistance mechanisms

In confronting the complexities of cancer, the interplay between drug resistance mechanisms and the metabolic reprogramming of cancer cells underscores a critical aspect of therapeutic challenges (Sun et al., 2020). Traditional modalities such as surgery, chemotherapy, radiotherapy, immunotherapy, and targeted therapy represent significant advances, yet the shadow of resistance looms large, diluting their long-term efficacy (Rulli et al., 2019). Central to addressing this issue are P-gp/B1 inhibitors, which aim to counteract cancer's defense mechanisms. Various strategies were proven effective *in vitro*, such as competitive and non-competitive inhibition, disruption of ATP hydrolysis, and altering cell membrane lipid composition (Al-Akra et al., 2018). This effort to sensitize cancer cells to therapy is further nuanced by the recognition that metabolic alterations in cancer cells, including those mediating P-gp/B1 activities to towards resilience against conventional treatments (Meschini et al., 2000). Notably, agents such as verapamil, cyclosporine A, dexverapamil, valspodar, and tariquidar have shown promise *in vitro* and in clinical trials as chemosensitizers. However, their clinical application has been hindered by issues like poor selectivity, low potency, high toxicity, and unpredictable pharmacokinetic interactions (Yahanda et al., 1992). Research efforts are increasingly directed towards exploring natural products and their structural alterations to develop novel P-gp/B1 inhibitors, potentially enhancing their safety and effectiveness (Li et al., 2014; Yoshida et al., 2006). Despite these efforts, the *in vivo* efficacy and safety of these inhibitors remains to be fully established, underscoring the need for further investigation (Li et al., 2014).

Interestingly, P-gp/B1 also plays a role in cancer metabolism or vice versa. Elevated expression of MDR1 has been shown in variety of tumors as opposed to normal tissue samples and in cancer cell lines as opposed to healthy cell (Noonan et al., 1990). Evidence from *in vivo* study shows that tumors with altered metabolic profiles, compared to treatment-naive tumors, have increased P-gp/B1 activity (Viale et al., 2014). Additionally, MDR1 expression profiles have been shown to be different between surgical samples from primary and metastatic tumors; metastatic CRC samples having the higher expression of MDR1 (Micsik et al., 2008). Considering the changing MDR1 expression throughout progression of cancerous cells – each having unique metabolic adaptation – metabolic adaptation has a great impact of MDR1 expression. This suggests a complex interaction between drug efflux mechanisms and metabolic reprogramming. This synergy between metabolic changes and P-gp/B1 activity illustrates the need for a multifaceted approach in cancer therapy, one that encompasses not just the inhibition of efflux pumps but also the targeting of metabolic vulnerabilities.

The metabolic reprogramming of cancer cells offers fertile ground for therapeutic intervention, with *in silico* research revealing how shifts in metabolism are integral to cancer progression and resistance (Sun et al., 2020). Therapy-resistant tumors exhibit enhanced reliance on mitochondrial metabolism compared to treatment-naive counterparts *in vivo* (Viale et al., 2014). This observation has led to strategies aiming to inhibit specific metabolic pathways to stymie tumor growth. For instance, research on *in vivo* tumor models has shown that anti-angiogenic kinase inhibitors have been shown to amplify AMPK signaling in breast and lung cancer models, shifting metabolism towards an oxidative phenotype (Navarro et al., 2016). This metabolic shift renders cells more susceptible to oxidative metabolism inhibitors, highlighting the potential of metabolic intervention in conjunction with traditional therapies (Navarro et al., 2016).

Additionally, inhibiting metabolic enzymes and pathways, such as lactate dehydrogenase (LDH) and pyruvate dehydrogenase kinase (PDHK) is another strategy in cancer treatment (Boudreau et al., 2016; Michelakis et al., 2010). Drugs targeting these pathways, like metformin, have shown the ability to kill chemotherapy-resistant breast tumor stem cells *in vitro*, highlighting the therapeutic potential of targeting cancer metabolism (Janzer et al., 2014). In the broader context, effective cancer treatment strategies are increasingly focusing on understanding the molecular and metabolic mechanisms behind resistance. Various approaches are being explored, from using miRNAs and siRNAs to target autophagy-related genes, to developing BH3 mimetics to induce apoptosis in cancer cells (Selvakumaran et al., 2013; Sümbül et al., 2014; Zhang et al., 2021). However, integrating metabolic interventions, either by directly targeting metabolic enzymes or by modulating pathways influenced by P-gp/B1, is becoming a crucial part of this evolving strategy. As research advances, the potential to disrupt the metabolic adaptations of cancer cells, thereby sensitizing them to both existing and novel therapies, offers a promising path forward in the relentless fight against cancer.

## Concluding remarks

As we conclude this Review, we must explore the complex relationships between drug sequestration and MDR emergence. The MDR1 gene, which encodes P-gp/B1, a membrane-bound efflux transporter with the remarkable capacity to actively expel a wide variety of medicines out of the cells, is at the center of this link (Gericke et al., 2022). As essential parts of the ES, lysosomes are essential for drug sequestration because of their acidic environment and ability to accumulate weakly basic medicines (Colombo et al., 2014). Drugs, particularly chemotherapeutics, are sequestered within lysosomes by means unrelated to transporters or enzymes (Colombo et al., 2014). When the MDR1 gene is overexpressed in cancer cells, P-gp/B1 activity increases, which amplifies the drug's outflow from the intracellular environment. Because of this increasing efflux, prescription efficacy is waning, and multidrug resistance is starting to appear. Developing strategic approaches for overcoming the formidable challenge of multidrug resistance in cancer treatment requires a deep comprehension of the complicated interplay between drug sequestration, lysosomal complexity, and MDR1 gene regulation.

CRC cell lines are not only capable of metabolic plasticity to activate alternative energy pathways under nutrient stress to gain survival advantages. But also, they can enhance the P-gp/B1 expression as another survival mechanism when nutrients are scarce. In CRC cell lines, nutrient depletion can increase MDR1 expression and enhance drug sequestration within lysosomes, further increasing resistance (Güleç Taşkıran et al., 2024a). Addressing these nutrient-related factors is crucial for developing effective therapeutic strategies.

Innovative approaches have been prompted by the potential to mitigate drug resistance, especially regarding anthracyclines. Gong et al. aimed to reduce the intrinsic basicity of daunorubicin by making structural changes to the drug. The resulting daunorubicin derivatives significantly reduced lysosomal sequestration *in vitro*, as observed in two resistant cell lines (Gong et al., 2003). Remarkably, however, these changed compounds' durability and efficacy beyond *in vitro* evaluations are still unknown, providing a path for future research and possible therapeutic uses.

Our focus is on promising approaches to drug resistance that are derived from recent advancements in nanotechnology to combat drug resistance and advance cancer therapy. Liposomes, polymers, metals, and metal-oxide nanoparticles are examples of how nanomaterials are influencing new therapeutic paradigms (Doshi et al., 2015; Ghafari et al., 2020; Pandey et al., 2016; Yang et al., 2021). These nanocarriers function as vehicles for precisely delivering therapeutic drugs to specific areas while limiting effects on healthy tissues by utilizing the enhanced permeability and retention (EPR) effect in the tumor vasculature (Matsumura and Maeda, 1986). Some nanoparticles possess intrinsic characteristics that enable them to modify signaling pathways associated with autophagy regulation (Zhang et al., 2014a). Because of its dual purpose, nanotechnology is positioned as a treatment approach against a range of human cancers. These strategies are a significant advance in the fight against drug resistance and offer hope for more effective, targeted, and minimally invasive therapeutic approaches.

In conclusion, our Review highlights the MDR1 gene's role in drug resistance, the role of metabolic adaptation on MDR1 expression patterns by emphasizing lysosomal involvement in sequestration. Innovative strategies, such as structural modifications and nanotechnology interventions, offer promising avenues for combating multidrug resistance and advancing more effective cancer therapies.
